# The Transactions of the Illinois State Medical Society

**Published:** 1856-01

**Authors:** 


*The Transactions of the Illinois State Medical Society.*

The regular Annual Meeting of the Illinois State Medical Society, was held in Bloomington, during the first week in June last, and yet we have seen nothing of the printed transactions.

Are we to have them; or have the publishing committee decided the *matter* to be not worth the cost of printing? Or, are there no funds in the treasury? Will the Secretary give us some light on the subject? If we remember correctly, money was contributed at the last meeting, to the amount of about fifty dollars, to be offered as a premium for the best essay on some medical subject, and a committee was appointed to receive and examine such essays as should be offered for such prize. If we mistake not, Dr. W. B. Herrick, of Chicago, was made chairman of that committee. It is certainly time that said committee should issue to the profession of the State some *notice*, if they wish to receive any essays before the next Annual Meeting of the State Society. We hope there will be no further delay in regard to these matters.

